# Survey on Tunisian Dentists' Anti-Inflammatory Drugs' Prescription in Dental Practice

**DOI:** 10.1155/2021/6633870

**Published:** 2021-01-31

**Authors:** Line Berhouma, Amira Besbes, Abdellatif Chokri, Jamil Selmi

**Affiliations:** ^1^Department of Oral Medicine and Oral Surgery, University Dental Clinic, 5019 Monastir, Tunisia; ^2^Medical and Molecular Parasitology and Mycology Laboratory, LR12ES08, 5019 Monastir, Tunisia

## Abstract

Dentists prescribe several types of drugs such as anti-inflammatory medicines in their practice in order to manage pain. An adequate knowledge of anti-inflammatory drugs' characteristics is mandatory for a reasonable prescription to ensure patients safety. The study aimed to describe dentists' anti-inflammatory drugs prescription in dental practice. *Materials and Methods*. This study was conducted on independent practice dentists working in the region of Tunis. A questionnaire was made on “Google forms” and sent to all of them via personal emails. The questionnaire included demographic data and 13 questions about anti-inflammatory medicines: indications and contraindications, the side effects, and their prescription in dental practice. Data analysis was performed on SPSS software version 20.0 (trial version), using the *χ*2 test for statistical analysis. *Results*. Two hundred dentists participated to the survey. The female gender was predominant (70%). More than half of the responders were recently graduated and working in their own dental offices. The present study showed that 60% of dentists rarely prescribe anti-inflammatory drugs. Ibuprofen was prescribed by 82% of the dentists. Next came dexamethasone acetate (68.2%). The most frequent indication was postoperative pain (65%). Gastric problem was found to be the most mentioned adverse effect (69%). Thus, 72% of the dentists prescribed proton pump inhibitors with AI. *Conclusion*. According to this study, dentists have a lack of knowledge and awareness about some contraindications, side effects, and drugs interactions. Thus, knowledge updating, practices assessment, and continuous education are always required to avoid drug iatrogenesis.

## 1. Introduction

Anti-inflammatory drugs are a widely used therapeutic class due to their antipyretic, analgesic, and anti-inflammatory activities.

The most prescribed classes of drugs in the dental practice are antibiotics, analgesics, antiseptics, and anti-inflammatory drugs (AIDs).

Dentists prescribe both steroids and nonsteroidal anti-inflammatory drugs (NSAIDs) for many reasons: dental or orofacial pain, postoperative pain after endodontic procedures, oral and implant surgery, or oral dermatologic diseases.

Each drug has its own characteristics, and each disease has its own medical treatment which influences drug indications and contraindications. Prescription of anti-inflammatory drugs (AIDs) must correspond to a standardized national or international prescription pattern. It should also be personalized for each patient according to his medical history to avoid possible interactions and undesirable effects.

AIDs have undeniable beneficial anti-inflammatory and analgesic properties. Nonetheless, the use of nonsteroidal AIDs can be associated with the higher risk of gastrointestinal, renal, and cardiovascular complications [1, 2]. Also, NSAIDs prescription in pregnancy cause potential adverse effects including labor prolongation, the constriction of the ductus arteriosus, and hemostatic and renal abnormalities in the fetus and neonate [3]. Moreover, short-term corticosteroid use is associated with generally moderate side effects such as cutaneous effects, electrolyte abnormalities, hypertension, hyperglycemia, and other systemic effects [4].

As for all the drugs, any error in AIDs prescription may introduce adverse effects and potential drug-drug interaction [5].

There are many studies assessing the prescription of antibiotics in the dental practice. This is probably in order to monitor the risk of the bacterial resistance emergence [6].

However, few studies assessing practices of dentists toward prescription of AIDs in Tunisia or elsewhere were published. Dentists must have the adequate knowledge for indications of AIDs, their pharmacologic characteristics, and their side effects and potential interactions. Thus, surveys concerning prescribing are needed for dentists to have an overview of their knowledge and their awareness in order to improve rational drug therapy and patients' safety.

In this context, this study aimed on describing dentists' anti-inflammatory prescription in dental practice.

## 2. Materials and Methods

This cross-sectional pilot study was performed on independent practice dentists practicing in the region of Tunis, capital of Tunisia, during the period between May and July 2019.

As Tunisian university courses are presented in French, the questionnaire was formulated in French, with reference to the relevant literature. It included 13 questions (Appendix) about dentists' knowledge of anti-inflammatory drugs and its use in dental practice: the prescribed molecules, the frequency of prescription, contraindications and their awareness about AIDs side effects, drugs interactions, and self-medication among patients. The internal consistency of the questionnaire was assessed, and Cronbach alpha coefficient was equal to 0.72.

The self-administrated questionnaire was entered on “Google forms” and randomly sent to the dentists via personal emails. The principal investigator explained that this study was undertaken by the third author in fulfillment of the requirements for the degree of Doctor in Dental Medicine in the Faculty of Dental Medicine of Monastir.

All the dentists practicing in the region of Tunis, the capital of Tunisia, were included in this study. They were 800 in 2019, according to the Regional Council of the Order of Dentists.

We considered that participants gave an informed consent to the participation since they responded to the survey.

Data obtained from the questionnaire were also transferred to SPSS software 20.0 (trial version), where descriptive statistics were analyzed. The *X*^2^ test was used to analyze qualitative data. The significance level was set at 95%, and the maximum acceptable difference was 5%.

## 3. Results

A random sample of 200 dentists practicing in Tunis participated to the survey. The response rate was then 25%. The mean age was 35±9 years. The female gender represented 70% of them. More than half of the responders were recently graduated and working in their own dental offices. The present study showed that 60% of dentists rarely prescribe AI drugs. Ibuprofen was prescribed by 82% of the dentists. Dexamethasone acetate was prescribed by 68% of them.

Sixty-five percent of the participants prescribed AIDs to treat postoperative pain. Half of them prescribed those medicines for oral infections with dental origins such as cellulitis and abscess. Some of them prescribed AIDs to treat temporomandibular joint pain (33%).

Most of the dentists (61%) prescribed AIDs for a short-term period (less or equal to 3 days). According to them, the most cited contraindications were severe gastric disease (98%) followed by pregnancy (30%). Only few dentists cited allergic background, kidney failure, hepatic failure, heart failure, and HTA and respiratory impairment with, respectively, 13%, 16%, 7%, 9%, and 6%.

Regarding side effects, dentists cited mainly gastric irritation, kidney failure, allergic reactions, and hepatic failure in, respectively, 69%, 16%, 10%, and 6.5%.

According to this study, 82% of the participants declared that they were aware of gastric irritation related to AIDs and 72% of them prescribed proton pump inhibitors with AIDs with statistically significant association (*p* < 0.05).

When asked about drugs interactions, 76% of dentists reported that they took into account risks of drugs interactions when prescribing AIDs. Majority of them did not prescribe AIDs with other AIDs (98%). About 70% of them avoided the association with antiplatelet agents and anticoagulants. Sixty percent avoided the association with methotrexate. About half of them avoided the association with phenytoin, diuretics, and lithium, and 33% did not associate AIDs with antidiabetics. The other dentists collaborated with the treating doctor of the patient in order to adjust the dosage according to the doctor's recommendations.

Most of the dentists declared that they were aware that patients may practice self-medication before doctor's visiting (97%). Most of them asked the patients if he/she took an AI drug before prescription, discussed AI drugs' adverse effects and contraindications, and explained the inherent risks in taking medication without a doctor or pharmacist advice (95%), AIDs with statistically significant association (*p* < 0.05).

## 4. Discussion

Dentists prescribed AIDs in different conditions during their dental practice. Most odontogenic or postoperative pain can be relieved by nonsteroidal anti-inflammatory drugs.

The use of AIDs has been associated with the potential for the development of drug misuse. Therefore, the present study was conducted to give an overview about this practice and the knowledge about AIDs indications, side effects, interactions with other medicines, and the self-medication among patients.

We noted that dentists shortened the list of AID and only prescribed few molecules, their generics, or their brand name among a multitude of available medicines. This is in agreement with the P-drugs concept, wherein doctors select their personal medicines with which they are more familiar and prescribe them regularly as a priority choice for given indications [7, 8].

Our study showed that ibuprofen was the most frequently prescribed NSAIDs. This result is in accordance with previous studies [6, 9–15].

Ibuprofen use was a subject of controversy. In fact, Sepheri et al., 2018, reported that there were no studies showing that ibuprofen is more efficient than other NSAIDs [11]. Nevertheless, it was reported that this molecule was better tolerated [7]. It has been suggested that this molecule is the most efficient to treat dental pain [16, 17] and its action duration and side effects, especially gastrointestinal ones are less than other NSAIDs [16, 18].

Additionally, our study showed a variety of other NSAIDs prescription such as piroxicam and naproxen. Here, it may be noted that these drugs were not recommended for dental pain management because of their severe adverse effects [17, 19].

The present finding indicated that dexamethasone was the second AID and first steroidal AID prescribed by dentists. This result is also in agreement with the result of Hashemipour in 2019 [15]. Furthermore, clinical trial research studies are needed to assess and compare the efficacy of different AIDs in management of dental pain.

Dentists prescribe AID for different reasons but mainly to treat or to prevent pain. Orofacial pain related to the teeth is the most described form [11]. According to the present study, 65% of dentists prescribed AID to prevent or to treat postoperative pain. It was reported that the preoperative dose of NSAIDs may decrease the number of postoperative analgesics required [20].

Postoperative includes endodontic procedures and oral surgery such as teeth removing. Patients suffer from mild to severe pain and consequently need medication.

One of the reasons for prescription in this study was infection which is a source of pain.

None of the participants mentioned the use of AIDs in the neuralgia relief especially for the inferior alveolar nerve.

It is important to recognize the type and the origin of this latter to carry out the appropriate treatment and to provide the accurate prescription [12].

Dentists' knowledge came from university courses, scientific manifestations, and medical representatives. It is important to have the proper pharmacological knowledge in order to avoid prescription errors.

Most of the participants reported that they prescribed AIDs for a short period. In this case, it generally corresponded to the cited indications which included postoperative pain. AIDs should be prescribed for a precise period after which operative site should be reassessed if the pain persists.

Oral mucosal disease treatment was also reported by the dentists. Treatment of such dermatologic disease usually requires steroids for an intermediary period.

Whatever the indication of AIDs, the treatment duration should also conform to the rules of good practice. Therefore, in order to avoid potential complications, it is essential to raise awareness among the dental practitioners of the appropriate indications and dosage regimen of specific drugs [21].

Most of the participating dentists (91%) recognized gastric irritation as a side effect.

Minor side effects of AID are related to gastric toxicity and irritation. Gastrointestinal adverse events mentioned by the participants were nausea, vomiting, dyspepsia, abdominal pain, peptic ulcer disease, gastrointestinal bleeding, diarrhoea, constipation, and gastric reflux. Seventy-four percent of them prescribed proton pomp inhibitors in association with AID to minimize this effect. Prescription of gastroprotective agents, such as histamine type-2 (H2) blockers and proton pump inhibitors, is common in the world [22].

Serious side effects include renal impairment, hepatic failure, cardiovascular events, neurologic effects, respiratory dysfunction, hematological abnormalities, and allergic reactions.

It seemed that there was a lack of knowledge about systemic side effects. In fact, only few study participants cited allergic reactions, kidney failure, hepatic failure, respiratory impairment, osteoporosis, salt and water retention, oedema, hyperglycemia, cardiovascular risks, immunosuppression, and infection exacerbation.

In this study, the majority of the participants declared that they prescribed AID for short time therapy. Corticosteroids' short-term treatment is generally safe [23]. Nevertheless, mild side effects, including cutaneous effects, electrolyte abnormalities, hypertension, hyperglycemia, pancreatitis, hematologic, immunologic, and neuropsychologic effects and clinically significant side effects may occur [4].

It should be emphasized that even short time treatment with NSAID can be associated with an increased risk of cardiovascular events, leading to death according to Hoxha et al., 2020 [24]. Thus, duration of the treatment should be respected.

Dentists must have the adequate knowledge for clinical indications of AID. Thus, educational programs concerning prescribing guidelines are needed for dentists to improve rational drug therapy and patients' safety.

Most of the participants were aware about drugs interactions with AIDs. They referred to the treating doctor of their patients to ask about the safety of the association between antiplatelet agents, anticoagulants, methotrexate, and AIDs. Most of them avoided some associations. However, avoiding such prescription may not be the best solution to manage the pain. Therefore, it is important to measure the benefits and the risk of such associations. Drug interactions lead to toxicity or therapeutic failure [25].

One of the methods to minimize drugs interactions is to get informed about the associated medication. Such information is necessary to adapt or to avoid some prescriptions.

Dentists must ask patient about his medical history and current medication in order to reduce drug interactions. They may use alternative drugs where indicated or reduce the dose of the AIDs if possible. Patients and treating doctors should provide updated information, and a thorough AIDs intake should also be recorded and clearly documented by the dentist.

Tunisia is a developing country where patients are not adequately aware about self-medication risks. Data about self-medication are not available, but overall, people and even medical and dental students practice self-medication. They buy drugs directly from pharmacies, which may increase the risk of health hazards. This can be due to restricted financial resources, unwillingness to visit the dentist, poor sociocultural background ignoring self-medication risks, considering pain as a minor illness, and sometimes busy time.

Patients should be routinely asked and warned about the hazards of receiving AIDs from pharmacists or even from other physicians because inappropriate or excessive drug prescription not only leads to financial loss for patients but also causes adverse effects and serious complications. Health decision makers should work cooperatively with pharmacists and doctors to adopt the necessary measures allowing control AIDs sales with a view to minimizing self-medication.

This study gave an overview of dentists' practices. However, it had some limits.

It was a pilot study where the sample size was small. This study merits to be reconducted with larger sample. As with any self-administered survey, the answers given by participants may not reflect real actual practices and attitudes.

Moreover, our study analyzed the prescription of AIDs in general. We suggest that questions should be organized in two categories: questions about nonsteroidal AI drugs and corticosteroid AI drugs separately.

Also, we included in our study both general dentists and specialist dentists (mainly oral surgeons). Thus, this may affect the prescription type, prevalence, and indications; so, we suggest that prescription practices of both groups of dentists should be evaluated separately in future studies.

It would have been helpful if our data assessed knowledge with precise scores including, for example, patients' main diagnosis and complications for which drugs were prescribed.

To the best of our knowledge, this is the only study in Tunisia that focus on dentists' AI medicines prescription. Further studies with prescription patterns are needed to assess knowledge, awareness, and practices of Tunisian dentists.

## 5. Conclusion

According to this study, dentists prescribed AIDs when indicated for temporomandibular pain and postoperative pain especially after endodontic procedures or wisdom teeth removal. They were aware of certain side effects such as gastric irritation and iatrogenesis for pregnancy. They were also aware of self-medication among patients and tried to prevent it. However, pitfalls were observed in areas of systemic complications.

Based on our findings, we suggest that continuous education programs are mandatory to insist on the risks of AIDs, their contraindications, and assist dentists to choose the appropriate molecule with respect to the national or/and international guidelines for AID prescription. Moreover, it is necessary to raise public awareness of self-medication risks in order to avoid health hazards and risk of legal actions.

## Data Availability

The data used to support the findings of this study are included within the article.

## Appendix

  First part:
 (1) Age
 (2) Gender
 (3) Number of working years
 (4) General practitioner or specialist
  Second part
 (I) Assessment of prescribing habits
  Question 1: In your current practice, how often do you prescribe AIDs
  1. Always 2. Frequently 3. Rarely 4. Never
  Question 2: Do you associate AIDs with an antibiotic
  1. Always 2. Frequently 3. Rarely 4. Never
  Question 3: Which AIDs do you usually prescribe
  Active principle Example
  Mefenamic acid PONSTYL®
  Niflumic acid NIFLURIL®
  Tiaprofenic acid SURGAM®, FLANID®
  Ketoprofen BIPROFENID®, KETOFEN®
  Celecoxib ADYBREX®, ALGIBREX®, CELEBREX®, INFLACOX®
  Diclofenac DICLOFEN®, VOLTARENE®
  Etodolac LODINE®
  Ibuprofen BUPROFEN®, DOLVEN®, GELUFENE®, IBUCARE®, NOVAFEN®
  Piroxicam CYCLADOL®, PIROXEN®, ROXAM®
  Naproxen NAPROX®, APRANAX®, NOXEN®
  Prednisolone sodium metasulfobenzoate SOLUPRED®
  Prednisone CORTANCYL®
  Betamethasone disodium phosphate BETNESOL®
  Dexamethasone acetate DECTANCYL®, UNIDEX®
  Question 4: In what situations do you use AIDs
 (1) Mucous ulcerations
 (2) Temporomandibular joint pain
 (3) Inflammatory manifestations of infections
 (4) Postoperative pain
 (5) Others, specify
  Question 5: Overall (on all of your prescriptions) when you prescribe a AIDs, do you use them for periods
    (1) Short [<3 days] 2. Intermediate [between 3 and 7 days] 3. Long [> 7 days]
 (II) Assessment of precautionary employment situations
  Question 6: When you prescribe AIDs, do you take into account the contraindications
     Yes   No
  If so, why
  ................................................................................................................................
  .....................................................
  Question 7: When prescribing AIDs, do you take their side effects into account ?
     Yes   No
  If yes, which one
  ................................................................................................................................
  Question 8: Do you prescribe PPIs (proton pump inhibitor) when you prescribe an AID   Yes   No
  If so, in which situations
  ...................................................................................................................................
  Question 9: Do you take into consideration drugs interactions?
  If yes what are the different molecules that may have interactions with AIDS?
  Question 10: Do you prescribe AIDs in combination with
  Other NSAIDs Yes Yes, by reducing the dose No
  (ii) Antiplatelet agents exp: CLOPIDOGREL® Yes Yes, by reducing the dose No
  (iii) Oral anticoagulants exp: SINTROM® Yes Yes, by reducing the dose No
  (iv) Methotrexate exp: NOVATREX® Yes Yes, by reducing the dose No
  (v) Oral antidiabetics exp: GLUCOPHAGE®
  (vi) Yes Yes, by reducing the dose No
  (vii) Phenytoin exp: DIHYDAN® Yes Yes, by reducing the dose No
  (viii) Diuretics exp: LASILIX® Yes Yes, by reducing the dose No
  (ix) Lithium
  Yes Yes, by reducing the dose No
 (III) Assessment of the management of self-medication
  Question 11: Do you look for a possible self-medication before any prescription of AIDs
     Yes   No
  Question 12: Are you doing prevention to limit self-medication in your patients
     Yes   No
  Question 13: Do you discuss with your patients the potential risk of self-medication with AIDs
     Yes   No
